# Comparative outcomes of endovascular versus synthetic bypass for isolated superficial femoral artery lesions: A 3-year single-center cohort study

**DOI:** 10.1371/journal.pone.0361589

**Published:** 2026-11-05

**Authors:** Changsung Han, Gwon-Min Kim, Jaewon Choi, Young Jin Tak, Chiseung Lee, Chung Won Lee, Miju Bae, Il Jae Wang, Up Huh

**Affiliations:** 1 Department of Thoracic and Cardiovascular Surgery, Pusan National University School of Medicine, Biomedical Research Institute, Pusan National University Hospital, Busan, Republic of Korea; 2 Medical Research Institute, Pusan National University School of Medicine, Yangsan, Republic of Korea; 3 Department of Family Medicine, Pusan National University School of Medicine, Biomedical Research Institute, Pusan National University Hospital, Busan, Republic of Korea; 4 In Silico Medicine Lab, Biomedical Research Institute, Pusan National University School of Medicine, Busan, Republic of Korea; 5 Department of Emergency Medicine, Pusan National University School of Medicine, Biomedical Research Institute, Pusan National University Hospital, Busan, Republic of Korea; Ataturk University Faculty of Medicine, TÜRKIYE

## Abstract

Peripheral artery disease (PAD) ranges from intermittent claudication to critical limb‑threatening ischemia (CLTI), with <3% of intermittent claudication cases progressing to CLTI. This study aimed to compare the outcomes of percutaneous transluminal angioplasty with stenting (PTAS) and synthetic graft femoropopliteal bypass (FPB) in a homogeneous PAD cohort. A subgroup analysis of TransAtlantic Inter-Society Consensus (TASC) B and C lesions, which lack standardized treatment algorithms, was also performed. We retrospectively analyzed 88 patients with no prior history of vascular interventions in the same limb who were treated for isolated superficial femoral artery lesions between 2016 and 2020 (29 underwent PTAS and 59 underwent FPB). Primary outcomes were reintervention, limb amputation, mortality, and vessel patency (primary, assisted primary, and secondary) over a 3‑year follow‑up. Subgroup analysis was performed to assess differences in treatment outcomes. No statistically significant differences were detected in reintervention, limb amputation, mortality, or patency between the two treatments. For loss of primary patency, the hazard ratio (HR) for FPB versus PTAS was 1.89 (95% confidence interval [CI], 0.76–4.69; log-rank *p* = 0.146) and 1.23 (95% CI, 0.45–3.38) after adjustment for lesion length and TASC grade. In the TASC B and C subgroup, no significant differences were observed in primary, assisted primary, or secondary patency (log-rank *p* = 0.809, 0.302, and 0.135, respectively). The study was underpowered to detect modest effects (post-hoc power for primary patency, 34.4%). In this small single-center cohort, no statistically significant difference in 3-year outcomes was detected between PTAS and FPB for isolated superficial femoral artery lesions; clinically meaningful differences cannot be excluded, and the findings should be regarded as hypothesis-generating.

## Introduction

Peripheral artery disease (PAD) is characterized by stenosis or occlusion of the arteries supplying the limbs, resulting in reduced blood flow and, ultimately, tissue necrosis. It affects over 200 million people worldwide, and its prevalence rate has increased by 25% over the last decade, with approximately 20% of affected individuals aged >80 years [1]. PAD ranges from intermittent claudication to critical limb‑threatening ischemia (CLTI), with <3% of intermittent claudication cases progressing to CLTI [1]. Without appropriate treatment, approximately 25% of patients with CLTI undergo limb amputation within 1 year of diagnosis, and the mortality rate ranges from 20–26% [2]. The risk increases further with tissue loss [2], underscoring the importance of early diagnosis and timely treatment to reduce amputation rates [2,3].

Femoropopliteal disease is the most frequent manifestation of atherosclerosis in patients with symptomatic PAD. Femoropopliteal disease can be managed with multiple revascularization strategies; however, determining the optimal treatment modality for each patient remains challenging. The Trans‑Atlantic Inter‑Society Consensus (TASC) II classification is widely used to guide lesion‑specific treatment strategies, categorizing them into grades A–D based on lesion length and the degree of stenosis/occlusion, with corresponding treatment recommendations [3]. However, treatment decisions in clinical practice are influenced by the anatomical characteristics of the lesion and the patient’s overall health, vascular status, and preferences of the patient and the operator [2]. Endovascular treatment for femoropopliteal TASC A and B lesions generally demonstrates favorable patency and reintervention rates. Recent advances in endovascular techniques have expanded their applicability, and TASC C and D lesions—historically treated with surgical bypass—are increasingly being considered for endovascular approaches [3,4]. The most recent European Society of Cardiology guidelines recommend endovascular treatment for superficial femoral artery (SFA) lesions up to 25 cm in length, whereas patients with longer lesions may benefit from venous bypass [5]. Given the higher perioperative risk in many patients, an endovascular approach may be advantageous in minimizing adverse events [5].

Rapid restoration of limb blood flow is essential in the management of PAD [2]. However, studies directly comparing optimal first‑line treatments are limited. For isolated SFA lesions, a growing clinical consensus favors an “endovascular‑first” approach; however, robust supporting evidence remains lacking [6,7]. Large‑scale trials, such as BASIL‑1, BASIL‑2, and BEST‑CLI, have included heterogeneous anatomical lesions and primarily focused on CLTI, potentially limiting the applicability of their findings to isolated SFA disease [6–8]. In addition to the choice of initial treatment modality, maintaining long‑term vessel patency often requires timely assisted primary or secondary interventions when restenosis occurs. Although institutional ability to provide structured follow‑up and prompt secondary intervention has been reported as a possible influencing factor in achieving long‑term outcomes, it has not been a primary focus of most previous comparative studies. The present study aimed to compare the outcomes of endovascular treatment and synthetic graft bypass as primary revascularization strategies in patients with treatment-naïve isolated SFA lesions and identify factors to relevant to selecting the optimal treatment strategy for maintaining patency. Given its retrospective single-center design, this study was intended to be hypothesis-generating.

## Materials and methods

### Study design

This retrospective study analyzed electronic medical records of patients diagnosed with PAD and treated at our institution between January 2016 and December 2020, focusing on those with lesions confined to the SFA. The study was approved by the Institutional Review Board of Pusan National University Hospital (IRB No. 2505-016-151; dated: May 30, 2025). Medical records and archived samples were accessed for research purposes between May 30, 2025, and March 31, 2026. The authors had access to medical records containing identifying information during the initial data collection phase. However, all personal identifiers were removed from the dataset extracted for analysis. Consequently, the authors did not have access to information that could identify individual participants during or after the data analysis and results generation process. The requirement for informed consent was waived by the institutional review board owing to the retrospective study design. This study adheres to the Strengthening the Reporting of Observational Studies in Epidemiology statement for cohort study reporting. All procedures were conducted in accordance with the ethical standards of the institutional research committee and the 1964 Helsinki declaration and its later amendments.

### Participants

Participants were enrolled according to predefined inclusion and exclusion criteria. Eligible patients were those who, on duplex ultrasonography, exhibited no suitable great saphenous vein for bypass and were diagnosed with SFA occlusive disease. In addition, participants were required to have no significant (≥50%) stenosis or occlusion in the proximal inflow vessels, and at least one patent distal runoff artery, including the posterior tibial artery, anterior tibial artery, or peroneal artery. Patients were excluded if they had a history of prior vascular interventions in the same limb, or if the target lesions involved non-SFA segments such as the iliac or popliteal arteries or extended into these vascular territories. Individuals who underwent endovascular procedures limited to balloon angioplasty, drug-coated balloon, or drug-eluting stent implantation, as well as those who received hybrid surgical procedures, were also excluded from the analysis. Fig 1 presents a flowchart of the inclusion and exclusion process.

**Fig 1:**
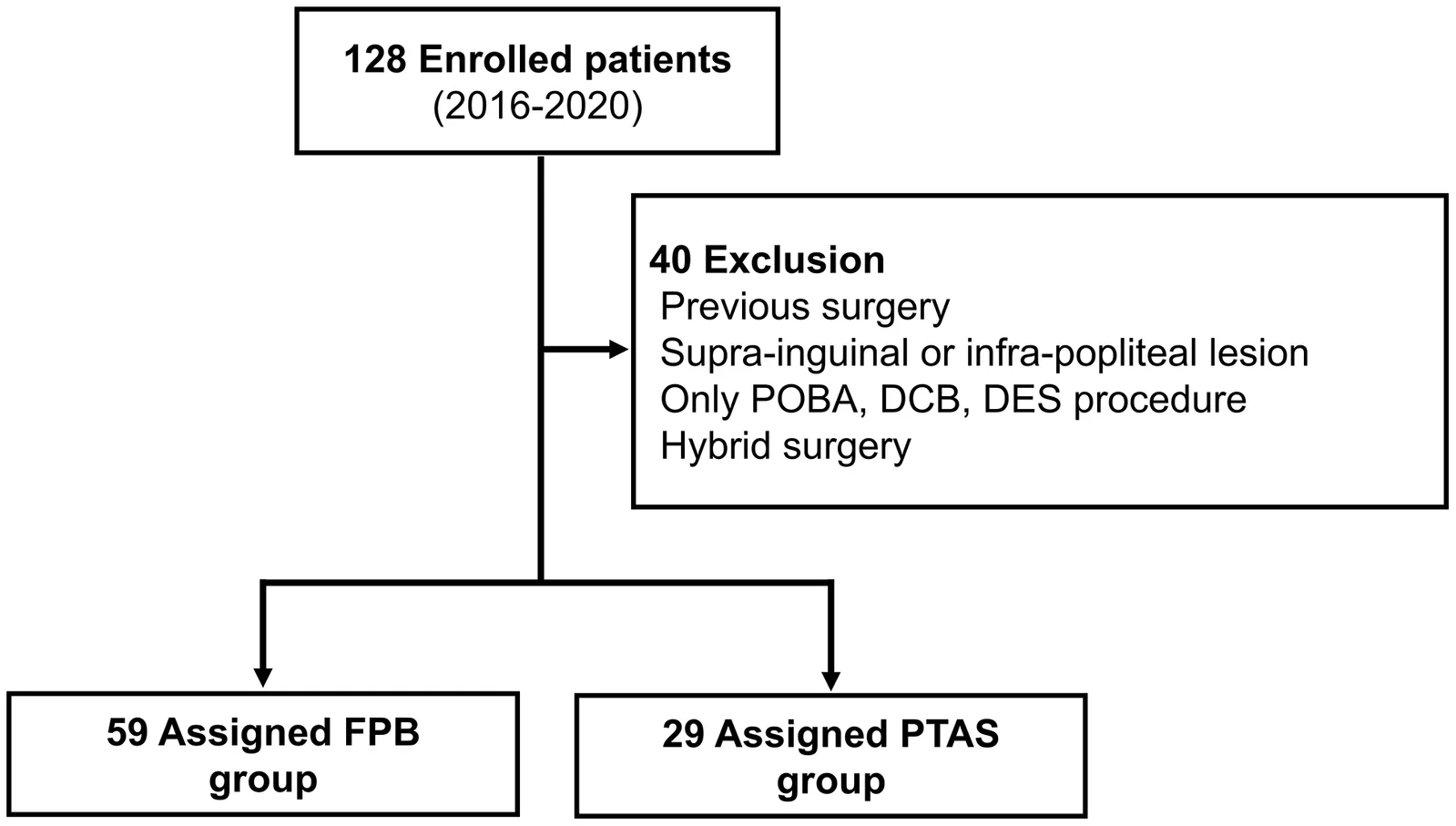
Inclusion/exclusion algorithm.

Treatment selection was based on lesion characteristics, comorbidities, and overall patient condition. Participants were divided into two treatment groups: endovascular intervention group (n = 29) and surgical synthetic graft bypass group (n = 59).

Endovascular treatment involved balloon angioplasty followed by bare‑metal stent placement. Surgical treatment involved a synthetic graft bypass from the femoral artery to the popliteal artery or distal SFA. The following clinical outcomes were compared between groups: reintervention rate, limb amputation rate, and overall mortality rate. Vessel patency was evaluated as primary, assisted primary, and secondary patency. All procedures were performed under local or general anesthesia by four experienced vascular surgeons and one interventional specialist.

### Intervention procedures

All patients were prescribed antiplatelet therapy according to current guidelines. Aspirin was administered to all patients as standard care. Dual antiplatelet therapy (aspirin and clopidogrel) was prescribed for patients undergoing percutaneous transluminal angioplasty with stenting (PTAS) and those undergoing synthetic graft bypass surgery.

### Endovascular treatment

Endovascular interventions were performed by an interventional radiologist or a vascular surgeon. All stents used were bare‑metal stent following pre‑dilatation with plain old balloon angioplasty. No drug-coated balloon or drug-eluting stent were deployed. The choice of stent placement was made selectively at the discretion of the treating physician.

Under local or general anesthesia, patients were placed in the supine position, and the operative site was prepared with Betadine and chlorhexidine draping. Depending on vascular anatomy, either the contralateral or ipsilateral common femoral artery was punctured under ultrasound guidance, and a 6–7 Fr sheath was introduced. A guidewire, with or without a shuttle catheter, was advanced across the lesion. Lesions were treated using a 6–7 mm plain old balloon angioplasty followed by a 7–8 mm bare‑metal stent for residual stenosis or occlusion. Hemostasis at the puncture site was achieved using a vascular closure device, and patients remained on bed rest for 4 h before resuming mobilization.

### Surgical treatment

For synthetic graft femoropopliteal bypass (FPB) surgery, patients without suitable autogenous great saphenous vein underwent bypass with a polytetrafluoroethylene synthetic graft. The common femoral artery and either the distal SFA or the popliteal artery were exposed. A 7-mm polytetrafluoroethylene graft was anastomosed end‑to‑side to the common femoral artery proximally and to the distal SFA or popliteal artery distally using 6‑0 Prolene sutures. The graft was tunneled subfascially with a tunneler. Skin closure and sterile dressing were performed at the end of the procedure.

### Postoperative surveillance

All patients underwent a standardized clinical and radiographic surveillance protocol. Physical examinations including palpation of peripheral pulses and assessment of Doppler sound, were performed at 2 weeks; 1, 3, 6 and 12 months postoperatively; and every 6–12 months thereafter. Re-intervention was considered when patients exhibited recurrent symptoms (Rutherford category ≥ 3) and Ankle–Brachial Index < 0.7 associated with significant restenosis or occlusion confirmed using computed tomography angiography. Follow-up duration was measured from The index procedure to the last documented assessment of graft or stent patency, and is reported as the median from the Kaplan-Meier method. Follow-up completeness was quantified using the follow-up index, the ratio of observed to potential follow-up time. Surveillance completeness at each time point was the proportion of eligible patients (those without a preceding loss-of-patency event) in whom patency status was documented.

Patency outcomes were defined in accordance with the reporting standards recommended by the Society for Vascular Surgery (SVS). Primary patency was defined as uninterrupted patency of the treated segment without any subsequent intervention, lost when imaging demonstrated recurrent stenosis ≥ 50% (defined by a peak systolic velocity ratio ≥ 2.5 on duplex ultrasound) or total occlusion. Assisted primary patency was defined as patency maintained by a reintervention (e.g., repeat angioplasty or thrombectomy) performed to treat a recurrent, hemodynamically significant stenosis before actual occlusion of the index segment. Secondary patency was defined as patency restored after complete occlusion of the target vessel via endovascular or surgical reintervention [9].

### Statistical analysis

Data are presented as mean ± standard deviation for continuous variables and proportions for categorical variables. Continuous variables were compared between the PTAS and synthetic FPB groups using the Mann–Whitney U test, and categorical variables were compared using the chi-square test or Fisher's exact test. Statistical significance was defined at *p* < 0.05. Kaplan–Meier survival curves were generated to analyze patients’ risk numbers and the standard error at different follow-up intervals for primary, assisted primary, and secondary patency outcomes. The number of patients at risk and 95% confidence intervals (CIs) are displayed on each survival curve. Kaplan–Meier curves were generated with compared using the log-rank test. The association between treatment and each patency outcome was estimated using Cox proportional-hazards regression and reported as hazard ratios (HRs) with 95% CIs. Univariable models estimated the crude effect; a multivariable model adjusted for lesion length, TASC grade, number of patent runoff vessels, CLTI status, age and diabetes; and, given the modest number of events, a parsimonious model adjusting for lesion length and TASC grade was used as the primary adjusted estimate. Because assisted primary and secondary patency had only 8 and 2 events, respectively, Kaplan–Meier estimates and log-rank tests were regarded as the primary analyses for these endpoints; Cox estimates for them are reported as exploratory only and were not interpreted. To address confounding by indication we additionally applied inverse probability of treatment weighting (IPTW) using stabilized weights from a logistic propensity-score model (covariates: lesion length, TASC grade, number of patent runoff vessels, CLTI status, age, and diabetes); weights were not truncated in the main analysis. Covariate balance was assessed with the absolute standardized mean difference (SMD), and an SMD < 0.10 was considered to indicate adequate balance. Because of the small sample size, sensitivity analyses were performed with weights truncated at the 1st/99th and 5th/95th percentiles and with a doubly robust model in which the IPTW-weighted Cox model was additionally adjusted for covariates that remained imbalanced after weighting. Statistical power for the primary-patency comparison was assessed with the Schoenfeld method (two-sided α = 0.05). Power was calculated as the total two-sided rejection probability, i.e., the sum of the rejection probabilities in both tails, rather than the one-tailed approximation. Using the observed number of events and allocation ratio, we calculated the minimum hazard ratio detectable at 80% power and the post-hoc power for the observed hazard ratio; for reference, the numbers of events and patients required to detect hypothetical hazard ratios of 1.5–3.0 were also calculated (S1 Table). Analyses were performed using SPSS (version 27.0; IBM Corp., Armonk, NY, USA) and Python 3.14 (lifelines and statsmodels packages).

## Results

### Participant demographics

This study included 88 patients: 29 (33.0%) in the PTAS group and 59 (67.0%) in the FPB group. No significant differences were observed between the two groups in baseline demographic variables, except for age and BMI. The mean age was significantly higher in the FPB group than in the PTAS group (71.29 ± 7.13 vs. 66.00 ± 7.65 years, *p* = 0.002), whereas the mean BMI was significantly lower in the FPB group (22.65 ± 2.58 vs. 24.04 ± 3.13 kg/m^2^, *p* = 0.030). No significant differences were observed in sex distribution, prevalence of diabetes mellitus, hypertension, smoking history, or other comorbidities between the groups (Table 1).

**Table 1:** Participant demographics and comorbidities.

Variables	Overall (n = 88)	PTAS (n = 29)	FPB (n = 59)	*p*-value
Sex (male)	74 (84.1)	26 (89.7)	48 (81.4)	0.317
Age (years)	69.55 ± 7.68	66.00 ± 7.65	71.29 ± 7.13	0.002
Height (cm)	162.84 ± 7.73	162.21 ± 6.59	163.15 ± 8.28	0.593
Weight (kg)	61.44 ± 9.71	63.55 ± 11.20	60.41 ± 8.80	0.154
BMI (kg/m^2^)	23.11 ± 2.84	24.04 ± 3.13	22.65 ± 2.58	0.030
DM	53 (60.2)	19 (65.5)	34 (57.6)	0.477
HTN	58 (65.9)	22 (75.9)	36 (61.0)	0.167
Smoking	49 (55.7)	12 (41.4)	37 (62.7)	0.058
CAOD	7 (8.0)	0 (0.0)	7 (11.9)	0.090
CVA	15 (17.0)	4 (13.8)	11 (18.6)	0.569
Hyperlipidemia	11 (12.5)	6 (20.7)	5 (8.5)	0.103
COPD	2 (2.3)	1 (3.4)	1 (1.7)	1.000
CKD	6 (6.8)	2 (6.9)	4 (6.8)	1.000

PTAS, percutaneous transluminal angioplasty and stenting; FPB, femoropopliteal bypass; BMI, body mass index; DM, diabetes mellitus; HTN, hypertension; CAOD, coronary artery occlusive disease; CVA, cerebrovascular accident; COPD, chronic obstructive pulmonary disease; CKD, chronic kidney disease. A p-value < 0.05 was considered statistically significant.

### Lesion characteristics

The distribution of Rutherford classifications differed significantly between the two groups. However, the proportion of patients with CLTI (Rutherford categories 4 and 5) was similar in both groups (17.2%, n = 5 in the PTAS group vs. 11.9%, n = 7 in the FPB group).

According to the TASC‑II classification, 58.6% of lesions in the PTAS group were classified as TASC A or B, whereas TASC C or D lesions predominated in the FPB group (83.1%, *p* < 0.05).

Participants in the PTAS group more frequently had ≥2 runoff vessels, with a significantly higher mean number of runoff vessels in the PTAS group than in the FPB group (2.07 ± 0.84 vs. 1.66 ± 0.69, *p* = 0.028). Lesion length was significantly greater in the FPB group than in the PTAS group (20.69 ± 6.91 vs. 12.19 ± 7.35 cm, *p* < 0.001). Moreover, the mean graft diameter used in the FPB group was larger than the mean stent diameter used in the PTAS group (7.36 ± 0.52 vs. 5.79 ± 0.62 mm, *p* < 0.001) (Table 2).

**Table 2:** Classification, lesion anatomy, and graft or stent size.

Variables	Category	Overall (n = 88)	PTAS (n = 29)	FPB (n = 59)	p-value
Rutherford classification	1	7 (8.0)	5 (17.2)	2 (3.4)	0.013
	2	15 (17.0)	3 (10.3)	12 (20.3)	
	3	54 (61.4)	16 (55.2)	38 (64.4)	
	4	9 (10.2)	2 (6.9)	7 (11.9)	
	5	3 (3.4)	3 (10.3)	0 (0.0)	
	CLTI (4, 5)	12 (13.6)	5 (17.2)	7 (11.9)	0.520
TASC II classification	A	10 (11.4)	10 (34.5)	0 (0.0)	<0.001
	B	17 (19.3)	7 (24.1)	10 (16.9)	
	C	38 (43.2)	10 (34.5)	28 (47.5)	
	D	23 (26.1)	2 (6.9)	21 (35.6)	
Number of patent runoff vessels	1	36 (40.9)	9 (31.0)	27 (45.8)	0.017
	2	34 (38.6)	9 (31.0)	25 (42.4)	
	3	18 (20.5)	11 (37.9)	7 (11.9)	
	No	1.80 ± 0.76	2.07 ± 0.84	1.66 ± 0.69	0.028
Lesion length		17.89 ± 8.09	12.19 ± 7.35	20.69 ± 6.91	<0.001
Graft or stent size		6.84 ± 0.93	5.79 ± 0.62	7.36 ± 0.52	<0.001

PTAS, percutaneous transluminal angioplasty and stenting; FPB, femoropopliteal bypass; CLTI, chronic limb-threatening ischemia; TASC II, TransAtlantic Inter-Society Consensus II. A p-value < 0.05 was considered statistically significant.

The median follow-up was 42 months in both the PTAS and FPB groups (reverse Kaplan-Meier method), with no significant difference in the distribution of follow-up time between them (*p* = 0.188). The follow-up index truncated at 36 months was 100.0% for both PTAS and FPB. Complete 36-month follow-up was achieved in 29/29 (100.0%) PTAS and 59/59 (100.0%) FPB patients, and no patient was lost to follow-up before 36 months (0/88, 0.0%; S2 Table). Surveillance imaging was complete in all eligible patients at every scheduled time point in both groups (100%; S3 Table). The number of patients at risk at each time point is shown beneath each Kaplan-Meier panel in Figs 2 and 3.

**Fig 2:**
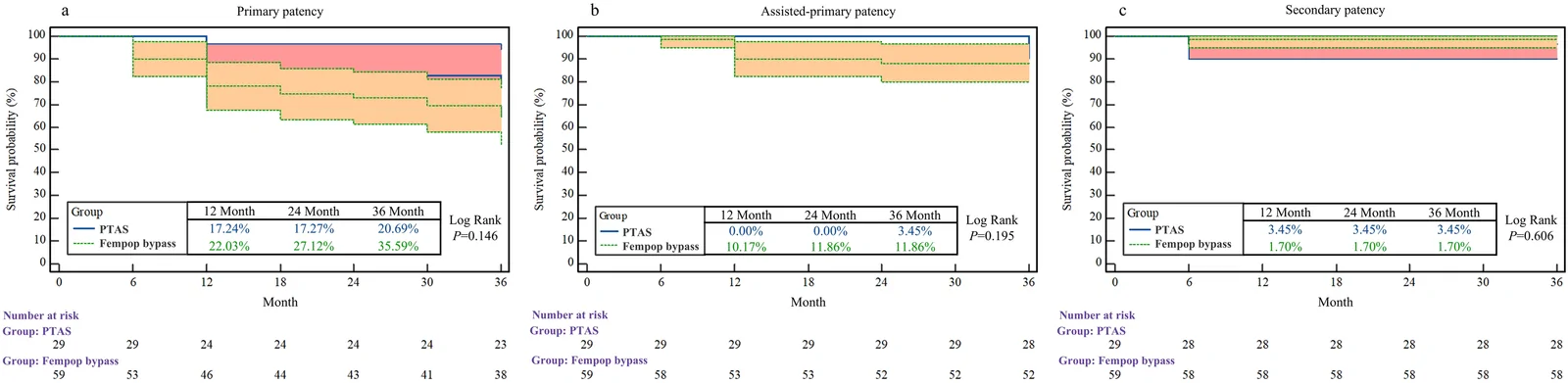
Kaplan–Meier Survival Curves for Primary, Assisted Primary, and Secondary Patency between PTAS and FPB Group. PTAS, percutaneous transluminal angioplasty with stenting; FPB, femoropopliteal bypass.

**Fig 3:**
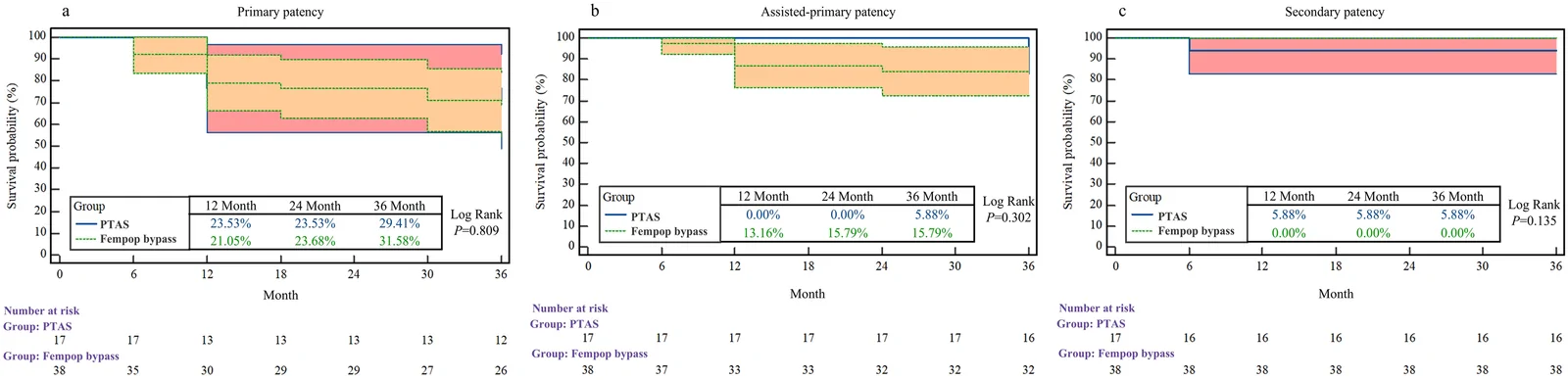
Kaplan–Meier Survival Curves for TASC-II B, C Classification Subgroup Analysis of Primary, Assisted Primary, and Secondary Patency. TASC, TransAtlantic Inter-Society Consensus; PTAS, percutaneous transluminal angioplasty with stenting; FPB, femoropopliteal bypass. Shaded areas indicate 95% CIs, and the numbers at risk are shown below each panel. Log-rank *p* = 0.809 (primary patency), *p* = 0.302 (assisted primary patency), and *p* = 0.135 (secondary patency).

**Treatment outcomes**

No statistically significant differences were observed between the PTAS and FPB groups in major clinical outcomes, including reintervention rate, limb amputation rate, and mortality. Technical success was achieved in 100% of cases in both groups. The FPB group had one case of vascular occlusion within 30 days and one limb amputation within 3 years; no statistically significant between-group difference in these outcomes was detected.

No significant differences were observed in postoperative complications, such as wound infection or bleeding. Kaplan–Meier survival analysis revealed no significant differences between the PTAS and FPB groups in primary patency (log rank *p* = 0.146), assisted primary patency (log rank *p* = 0.195), or secondary patency (log rank *p* = 0.606) (Fig 2).

### Subgroup analysis

A subgroup analysis was performed for patients with TASC B and C lesions (n = 55; PTAS, n = 17; FPB, n = 38) to assess the influence of lesion complexity on treatment outcomes, excluding those with TASC A or D lesions, as these have relatively clear treatment recommendations. No statistically significant differences in patency were observed between the PTAS and FPB groups (log-rank *p* = 0.809 for primary, *p* = 0.302 for assisted primary, and *p* = 0.135 for secondary patency; Fig 3). As an exploratory, descriptive observation, four of the five losses of primary patency in the PTAS group were recorded at the 12-month assessment (cumulative loss, 23.5% at 12 months and 29.4% at 36 months), whereas those in the FPB group were distributed across follow-up (21.1% and 31.6%, respectively). This difference in timing was not statistically significant (HR, 1.11; 95% CI, 0.39–3.15) and should not be regarded as a finding.

### Overall patency and adjusted analyses

In the unadjusted Cox model, the HR for loss of primary patency in the FPB group relative to PTAS was 1.89 (95% CI, 0.76–4.69) (*p* = 0.168). After adjustment the treatment effect attenuated toward the null and remained non-significant: the adjusted HR was 1.19 (95% CI, 0.42–3.39) (*p* = 0.742) in the full multivariable model and 1.23 (95% CI, 0.45–3.38) (*p* = 0.688) in the parsimonious model; the IPTW-weighted estimate was 2.00 (95% CI, 0.69–5.84) (*p* = 0.204). After weighting, the absolute SMD was <0.10 for TASC grade, number of runoff vessels, CLTI status, and age, but remained above 0.10 for lesion length (0.134) and diabetes (0.204) (S4 Table). The largest stabilized weight was 7.24, and the effective sample size of the PTAS group after weighting was 11.4. In sensitivity analyses, the HR was 1.74 (95% CI, 0.65–4.68) with weights truncated at the 1st/99th percentiles and 1.53 (95% CI, 0.60–3.94) with truncation at the 5th/95th percentiles, although truncation worsened covariate balance; in a doubly robust model additionally adjusted for lesion length and diabetes, the HR was 1.85 (95% CI, 0.66–5.20). All CIs included 1. For assisted primary patency (8 events) and secondary patency (2 events), the Kaplan–Meier estimates and log-rank tests are the primary results (Fig 2); the corresponding Cox estimates in Table 3 are exploratory only and are not interpreted.

**Table 3:** Crude and adjusted hazard ratios for loss of patency (FPB vs PTAS).

Endpoint (events)	Model	HR (95% CI)	p
Primary patency (events = 27)	Univariable	1.89 (0.76–4.69)	0.168
	Multivariable (full)¹	1.19 (0.42–3.39)	0.742
	Parsimonious²	1.23 (0.45–3.38)	0.688
	IPTW-weighted³	2.00 (0.69–5.84)	0.204
Assisted primary patency	Univariable	3.66 (0.45–29.78)	0.225
(events = 8; exploratory⁴)	Parsimonious²	2.95 (0.25–34.07)	0.387
	IPTW-weighted³	2.23 (0.28–17.73)	0.447
Secondary patency	Univariable	0.49 (0.03–7.82)	0.613
(events = 2; exploratory⁴)	Parsimonious²	0.14 (0.01–3.19)	0.221
	IPTW-weighted³	0.45 (0.03–7.31)	0.574

HR, hazard ratio; CI, confidence interval; IPTW, inverse-probability-of-treatment weighting; FPB, femoropopliteal bypass; PTAS, percutaneous transluminal angioplasty with stenting; EPV, events per variable. Reference group = PTAS. ^1^Adjusted for lesion length, TASC grade, number of runoff vessels, CLTI status, age, and diabetes. ^2^Parsimonious model adjusted for lesion length and TASC grade (primary adjusted estimate, respecting the EPV limit). ^3^Stabilized IPTW with robust standard errors; covariate balance before and after weighting is shown in S4 Table (absolute SMD < 0.10 considered adequate). ⁴Exploratory only; not interpreted because of the small number of events (the full multivariable model was not fitted for these endpoints). The primary analyses for these endpoints are the Kaplan–Meier log-rank tests (assisted primary patency, *p* = 0.195; secondary patency, *p* = 0.606). Cox proportional-hazards regression.

Given the observed 27 events and the 29:59 allocation, the analysis could detect only a hazard ratio of about 3.15 (or ≤0.32) at 80% power. The corresponding post-hoc power for the observed effect was 34.4% for the whole cohort and 5.5% for the TASC-II B/C subgroup (Table 4). For reference, the power to detect a hypothetical HR of 2.0 with the observed number of events was 39.5%, and approximately 241 patients (74 events) would have been required to detect this effect with 80% power (S1 Table).

**Table 4:** Post-hoc power and minimum detectable hazard ratio for the primary-patency comparison.

Cohort	PTAS n	FPB n	Events	Observed HR	Post-hoc power (observed effect)	Min. detectable HR (80% power)
TASC all	29	59	27	1.89	34.4%	3.15 (or ≤0.32)
TASC B/C	17	38	17	1.11	5.5%	4.35 (or ≤0.23)

HR, hazard ratio. Post-hoc power and minimum detectable HR computed for the primary-patency log-rank and Cox comparison at two-sided α = 0.05 from the observed number of events and the allocation ratio.

## Discussion

Despite numerous studies, universally applicable treatment guidelines remain elusive because of variability in lesion anatomy, patient comorbidities, and institutional expertise. In this study, we compared the outcomes of PTAS and synthetic FPB in patients with SFA lesions. A notable strength of our work was the dedicated subgroup analysis of TASC B and C lesions. TASC A lesions are typically managed with endovascular therapy, whereas D lesions are most often treated with surgical bypass. In contrast, TASC B and C lesions lack universally accepted treatment algorithms, and clinical decision-making for these intermediate-complexity cases remains highly variable [5]. Consequently, we performed a subgroup analysis on these categories.

Our results showed no statistically significant differences between PTAS and FPB in terms of limb salvage, mortality, reintervention, or patency. These findings are consistent with previous large-scale trials, such as BASIL‑1 and BEST‑CLI in patients with above‑knee disease without suitable vein conduit [6,8]. However, given the small sample size and limited statistical power, the absence of a statistically significant difference in our cohort should not be interpreted as evidence that the two strategies are equivalent. In our subgroup analysis of TASC B and C lesions, most losses of primary patency after PTAS were recorded by the 12-month assessment, whereas those after FPB were distributed over follow-up. However, cumulative loss of primary patency at 36 months differed little between the groups (29.4% vs. 31.6%), the difference was not statistically significant (log-rank *p* = 0.809), and this observation should be regarded as exploratory and descriptive only. If confirmed in larger studies, such a difference in timing could relate to procedural characteristics—PTAS achieves luminal gain through vessel dilation but is prone to early restenosis due to endothelial injury and inflammatory responses [10]. Restenosis after PTAS can occur at multiple unpredictable sites, requiring diligent monitoring and timely reintervention. In contrast, FPB creates a new conduit for blood flow, with restenosis usually localized to anastomotic sites, which are easier to detect and treat during follow-up [2,11].

The IPTW estimate for primary patency (HR, 2.00) did not move toward the null as the multivariable estimate did (HR, 1.19). These estimates are not expected to coincide: the multivariable Cox model estimates a conditional effect adjusted for a limited covariate set constrained by the events-per-variable rule, whereas IPTW estimates a marginal effect in a pseudo-population reweighted on the propensity score. In addition, the IPTW estimate was unstable in this small cohort. Because few PTAS patients had long or TASC D lesions, one PTAS patient with a 25-cm TASC D lesion and no loss of primary patency received a weight of 7.24, reducing the effective sample size of the PTAS group to 11.4, and lesion length and diabetes remained imperfectly balanced after weighting. Truncating extreme weights moved the estimate toward the null (HR, 1.53–1.74). The CIs of all estimates overlapped widely and included 1; therefore, none of the analyses supports a difference between the treatments.

In the absence of a demonstrated difference, the choice between PTAS and FPB for isolated SFA lesions, including TASC B and C lesions, should continue to be guided by established factors, such as patient comorbidities and lesion complexity. Institutional capacity for structured post-procedural follow-up may be a relevant contextual consideration, but its effect on outcomes could not be evaluated in this study. Furthermore, the applicability of these findings may depend on healthcare infrastructure. In lower-resource settings where intensive surveillance and timely reintervention are less accessible, any early restenosis after PTAS could carry a higher risk of unrecoverable occlusion, whereas the more predictable anatomical site of restenosis after synthetic FPB might be of practical relevance; this hypothesis requires evaluation in future studies.

We acknowledge that the endovascular treatment in our cohort (2016–2020) relied solely on plain old balloon angioplasty (POBA) followed by bare-metal stenting (BMS), which reflects the historical clinical practice of that period but limits the generalizability to contemporary standard. Recent trials have shown that drug-coated technologies reduce restenosis and target lesion revascularization compared with uncoated devices.

Specifically, the recent SirPAD randomized controlled trial (2026) [12], the largest RCT evaluating sirolimus-coated balloons (SCB) in infrainguinal disease, demonstrated a significant reduction in target lesion revascularization (TLR) and major adverse limb events compared to uncoated balloons. Furthermore, the SIRONA trial [13] established the non-inferiority of sirolimus-based balloons compared to paclitaxel-coated balloons in the femoropopliteal segment.

Had contemporary drug-eluting stents (DES) or drug-coated balloons (DCB) utilizing paclitaxel or sirolimus been applied to our PTAS cohort, the acute neointimal hyperplasia might have been suppressed, potentially reducing early restenosis after PTAS; therefore, our findings may not be applicable to contemporary drug-coated or drug-eluting platforms.

This study also has several limitations. First, this retrospective, single‑center analysis was constrained by a relatively small sample size, particularly within the TASC B/C subgroup (n ≈ 55), which limited our statistical power to detect clinically meaningful differences in 3-year primary patency and reintervention rates. Accordingly, the absence of statistically significant differences should be interpreted as insufficient statistical power (Type II error) rather than as evidence of equivalence between the two groups. However, the high homogeneity of our “isolated SFA-only” cohort partially mitigates this by minimizing anatomical confounding. Second, given the retrospective and non-randomized design, treatment allocation was based on physician discretion, which inherently introduces treatment-selection bias and baseline imbalances between the PTAS and FPB groups. Although we performed multivariable Cox regression and inverse probability of treatment weighting (IPTW) analyses to adjust for major clinical and anatomical confounders, residual unmeasured confounding (e.g., specific calcification patterns or vessel quality) cannot be entirely ruled out. In addition, the IPTW estimates were sensitive to a small number of patients with large weights, and lesion length and diabetes remained imperfectly balanced after weighting. The Cox estimates for assisted primary and secondary patency were based on very few events and are not interpretable. Furthermore, treatment paradigms continue to evolve with newer drug-coated or eluting devices and refined staging systems, such as the Global Limb Anatomic Staging System (GLASS), which may enable more precise, tailored interventions. Future adequately powered, multicenter prospective trials incorporating these contemporary modalities and staging systems will be essential to compare long-term patency between these strategies and optimize individualized treatment strategies.

## Conclusion

In this single-center cohort, no statistically significant difference in 3-year primary, assisted primary, or secondary patency, limb salvage, mortality, or reintervention was detected between PTAS with bare-metal stents and synthetic FPB for isolated SFA lesions. Given the limited sample size and post-hoc power, the study cannot exclude clinically meaningful differences, and the results should be interpreted as hypothesis-generating. Adequately powered prospective studies using contemporary endovascular devices are needed to confirm these observations.

## Supporting information

S1 Table Power and required sample size for hypothetical hazard ratios (primary patency). (DOCX)

S2 Table Follow-up characteristics by treatment group over the 36-month analysis horizon. (DOCX)

S3 Table Completeness of surveillance imaging at each scheduled follow-up time point, by treatment group. (DOCX)

S4 Table Covariate balance before and after inverse probability of treatment weighting. (DOCX)

## References

[1] Aru RG, Tyagi SC. Endovascular treatment of femoropopliteal arterial occlusive disease: Current techniques and limitations. Semin Vasc Surg. 2022;35(2):180–9. doi: 10.1053/j.semvascsurg.2022.04.010 35672108

[2] Conte MS, Bradbury AW, Kolh P, White JV, Dick F, Fitridge R, et al. Global vascular guidelines on the management of chronic limb-threatening ischemia. Eur J Vasc Endovasc Surg. 2019;58(1S):S1-S109.e33. doi: 10.1016/j.ejvs.2019.05.006 31182334 PMC8369495

[3] Norgren L, Hiatt WR, Dormandy JA, Nehler MR, Harris KA, Fowkes FGR, et al. Inter-society consensus for the management of peripheral arterial disease (TASC II). J Vasc Surg. 2007;45 Suppl S:S5-67. doi: 10.1016/j.jvs.2006.12.037 17223489

[4] Guez D, Hansberry DR, Gonsalves CF, Eschelman DJ, Parker L, Rao VM, et al. Recent trends in endovascular and surgical treatment of peripheral arterial disease in the medicare population. AJR Am J Roentgenol. 2020;214(5):962–6. doi: 10.2214/AJR.19.21967 32097027

[5] Vossen RJ, Vahl AC, Montauban van Swijndregt AD, Balm R. Clinical outcomes following invasive treatment of femoropopliteal artery disease: A retrospective single-center cohort study. J Vasc Interv Radiol. 2025;36(1):124-136.e1. doi: 10.1016/j.jvir.2024.08.030 39428061

[6] Farber A, Menard MT, Conte MS, Kaufman JA, Powell RJ, Choudhry NK, et al. Surgery or endovascular therapy for chronic limb-threatening ischemia. N Engl J Med. 2022;387(25):2305–16. doi: 10.1056/NEJMoa2207899 36342173

[7] Bradbury AW, Moakes CA, Popplewell M, Meecham L, Bate GR, Kelly L, et al. A vein bypass first versus a best endovascular treatment first revascularisation strategy for patients with chronic limb threatening ischaemia who required an infra-popliteal, with or without an additional more proximal infra-inguinal revascularisation procedure to restore limb perfusion (BASIL-2): an open-label, randomised, multicentre, phase 3 trial. Lancet. 2023;401(10390):1798–809. doi: 10.1016/S0140-6736(23)00462-2 37116524

[8] Adam DJ, Beard JD, Cleveland T, Bell J, Bradbury AW, Forbes JF, et al. Bypass versus angioplasty in severe ischaemia of the leg (BASIL): Multicentre, randomised controlled trial. Lancet. 2005;366(9501):1925–34. doi: 10.1016/S0140-6736(05)67704-5 16325694

[9] Jaff MR, White CJ, Hiatt JR, Fowkes GR, Dormandy J, Razavi MK. Reporting standards for endovascular treatment of lower extremity peripheral artery disease. J Vasc Surg. 2014;60(6):1680–98.

[10] Schillinger M, Exner M, Mlekusch W, Rumpold H, Ahmadi R, Sabeti S, et al. Vascular inflammation and percutaneous transluminal angioplasty of the femoropopliteal artery: Association with restenosis. Radiology. 2002;225(1):21–6. doi: 10.1148/radiol.2251011809 12354979

[11] Klinkert P, Schepers A, Burger DH, et al. Outcome of endovascular reintervention for significant stenosis at infrainguinal bypass anastomoses. J Vasc Surg. 2014;60(3):696–701. doi: 10.1016/j.jvs.2014.03.289 24810266

[12] Lortz J, Zeller T, Bausback Y, Krankenberg H, Lichtenberg M, Blessing E. Sirolimus-coated vs uncoated balloon angioplasty in infrainguinal peripheral artery disease (SirPAD): A randomized, multicenter, open-label, investigator-initiated trial. Lancet. 2026;407(10522):245–56.

[13] Zeller T, Blessing E, Lichtenberg M, Bausback Y, Krankenberg H, Sixt S, et al. Sirolimus-coated vs paclitaxel-coated balloons in femoropopliteal artery disease: the randomized SIRONA trial. N Engl J Med. 2025;393(12):1120–31.

