# Supplementary material for: Comparative outcomes of endovascular versus synthetic bypass for isolated superficial femoral artery lesions: A 3-year single-center cohort study
Source: PLoS One. 2026 Nov 5;21(11):e0361589. doi: 10.1371/journal.pone.0361589 (PMC13739158; doi:10.1371/journal.pone.0361589)
Supplement: Supplementary file 1 [file pone.0361589.s001.docx]

**S4 Table. Covariate balance before and after inverse probability of treatment weighting.**

| **Covariate** | **PTAS (before)** | **FPB (before)** | **SMD (before)** | **PTAS (IPTW)** | **FPB (IPTW)** | **SMD (IPTW)** |
| --- | --- | --- | --- | --- | --- | --- |
| Lesion length, cm (mean) | 12.19 | 20.69 | 1.208 | 17.77 | 18.74 | 0.134 |
| TASC II grade, score 1–4 (mean) | 2.14 | 3.19 | 1.237 | 2.95 | 2.98 | 0.040 |
| Patent runoff vessels, n (mean) | 2.07 | 1.66 | 0.539 | 1.68 | 1.74 | 0.080 |
| CLTI, % | 17.2 | 11.9 | 0.153 | 14.0 | 15.1 | 0.032 |
| Age, years (mean) | 66.00 | 71.29 | 0.725 | 69.60 | 70.10 | 0.069 |
| Diabetes mellitus, % | 65.5 | 57.6 | 0.163 | 49.2 | 59.3 | 0.204 |

Before, unweighted; IPTW, after stabilized inverse probability of treatment weighting; SMD, absolute standardized mean difference; PTAS, percutaneous transluminal angioplasty with stenting; FPB, femoropopliteal bypass; CLTI, chronic limb-threatening ischemia; TASC II, TransAtlantic Inter-Society Consensus II. Stabilized weights from a logistic propensity-score model including all covariates shown; weights were not truncated (range, 0.35–7.24; effective sample size after weighting, 11.4 for PTAS and 50.0 for FPB). An SMD <0.10 was considered to indicate adequate balance.
