# Supplementary material for: Comparative outcomes of endovascular versus synthetic bypass for isolated superficial femoral artery lesions: A 3-year single-center cohort study
Source: PLoS One. 2026 Nov 5;21(11):e0361589. doi: 10.1371/journal.pone.0361589 (PMC13739158; doi:10.1371/journal.pone.0361589)
Supplement: Supplementary file 2 [file pone.0361589.s002.docx]

**S2 Table. Follow-up characteristics by treatment group over the 36-month analysis horizon.**

| Characteristic | PTAS | FPB | Total |
| --- | --- | --- | --- |
| n | 29 | 59 | 88 |
| Median follow-up, mo (95% CI)ᵃ | 42 (42-42) | 42 (42-42) | 42 (42-42) |
| Observed follow-up, median (IQR), mo | 42 (42-42) | 42 (42-42) | 42 (42-42) |
| Follow-up index at 36 mo, %ᵇ | 100.0 | 100.0 | 100.0 |
| Complete 36-mo follow-up, no. (%)ᶜ | 29 (100.0) | 59 (100.0) | 88 (100.0) |
| Lost to follow-up before 36 mo, no. (%) | 0 (0.0) | 0 (0.0) | 0 (0.0) |

Abbreviations: PTAS, percutaneous transluminal angioplasty with stenting; FPB, femoropopliteal bypass; CI, confidence interval; IQR, interquartile range.
ᵃ Estimated by the reverse Kaplan-Meier method.
ᵇ Ratio of observed to potential follow-up time, both truncated at 36 months.
ᶜ Patency assessment documented at or beyond 36 months, or a patency endpoint reached before 36 months.
The distribution of follow-up time did not differ between groups (reverse Kaplan-Meier log-rank P = 0.188).
