# Supplementary material for: Comparative outcomes of endovascular versus synthetic bypass for isolated superficial femoral artery lesions: A 3-year single-center cohort study
Source: PLoS One. 2026 Nov 5;21(11):e0361589. doi: 10.1371/journal.pone.0361589 (PMC13739158; doi:10.1371/journal.pone.0361589)
Supplement: Supplementary file 3 [file pone.0361589.s003.docx]

**S3 Table. Completeness of surveillance imaging at each scheduled follow-up time point, by treatment group.**

| Group and measure | 6 mo | 12 mo | 18 mo | 24 mo | 30 mo | 36 mo |
| --- | --- | --- | --- | --- | --- | --- |
| PTAS, eligible for imaging, no.ᵃ | 29 | 29 | 24 | 24 | 24 | 24 |
| PTAS, patency status documented, no. | 29 | 29 | 24 | 24 | 24 | 24 |
| PTAS, completeness, %ᵇ | 100.0 | 100.0 | 100.0 | 100.0 | 100.0 | 100.0 |
| FPB, eligible for imaging, no.ᵃ | 59 | 53 | 46 | 44 | 43 | 41 |
| FPB, patency status documented, no. | 59 | 53 | 46 | 44 | 43 | 41 |
| FPB, completeness, %ᵇ | 100.0 | 100.0 | 100.0 | 100.0 | 100.0 | 100.0 |

Abbreviations: PTAS, percutaneous transluminal angioplasty with stenting; FPB, femoropopliteal bypass.
ᵃ Patients who had not reached a loss-of-primary-patency endpoint before the time point and therefore remained under surveillance.
ᵇ Proportion of eligible patients in whom patency status was documented at that time point.
Source of imaging counts: derived from coded observation times.
